# Supplementary material for: Perceived barriers for accessing international research funding among Latin American researchers
Source: PLoS One. 2026 Apr 20;21(4):e0343593. doi: 10.1371/journal.pone.0343593 (PMC13094988; doi:10.1371/journal.pone.0343593)
Supplement: S1 Table — (DOCX) [file pone.0343593.s001.docx]

Supporting information

**S1 Table. Focus group detailed subthemes and illustrative quotations.**

| **Themes** | **Subthemes** | **Incidents** | **Endorsement** |
| --- | --- | --- | --- |
| Strategies and good practices for navigating funding applications | Careful verification of eligibility | Ensuring alignment between the applicant’s profile, country of residence, and scope of the call was viewed as an initial critical step. Differences in scientific career structures across countries, particularly between Latin America and the Global North, were also noted as a challenge when interpreting eligibility criteria requirements. Participants highlighted the importance of attentively examining annexes and fine print to fully understand the requirements. | Participants 1, 4 |
|  | Early planning for administrative requirements | Carefully assess the time available to prepare the application, taking into account whether a pre-proposal or a full proposal is required, and the importance of early planning for administrative, ethical, and legal prerequisites such as certifications, institutional authorizations, or specific research permits. In general, all participants agreed that these requirements can be time-consuming and that their complexity often increases when working across institutions or countries. | Participants 1, 2, 3, 4, 5 |
|  | Adapting tone and language to the audience | Successful proposals were described as those calibrated to the expected reviewers’ background. Studying the profiles of potential reviewers was considered helpful to calibrate the level of technicality: *“If it’s a general grant, reviewed by people from many fields, you can’t make it too technical”* | Participant 5 |
|  | Peer review before submission | This was viewed as a good practice—one that not only improves the clarity of the proposal but also reduces disciplinary bias. However, trust, time, and goodwill were noted as prerequisites for this practice, which was reported to be more common among those with access to international networks | Participants 1, 2, 3, 4 |
|  | Collaborations and partnerships | Many participants agreed that applying with institutional or international partners increases the likelihood of success, though some also warned of the risks: *“Winning with the wrong partner can be a headache”* and *“Some big projects failed due to conflicts of interest.”* | Participants 3, 5 |
|  | Clear and compelling communication | All participants agreed and highlighted the importance of communicating project objectives clearly and simply. Overly complex or convoluted proposals were considered less likely to succeed, especially when reviewed under time constraints. One of them emphasized the need to simplify language and, also articulate a compelling narrative that aligns tightly with the call’s goals. | Participants 1, 2, 3, 4, 5 |
| Barriers and Structural Constraints Limiting Access to International Funding | Lack of clarity and transparency in national calls | A recurrent theme was the contrast between national and international calls. Participants highlighted that national calls were perceived as opaque, frequently tailored to specific groups, and entangled in heavy bureaucratic requirements. Evaluation criteria were described as vague, leaving researchers uncertain about how proposals would be judged. By contrast, international calls were generally perceived as more transparent and predictable: they provided clearer evaluation frameworks and, importantly, included dedicated contact people or helpdesks who responded quickly to queries. This availability of direct support during the application process was seen as a key difference that reduced uncertainty and frustration. | Participants 1, 3, 4 |
|  | Language and cultural barriers | Beyond English fluency, mastering rhetorical expectations of funders was seen as critical but challenging: *“You can’t write the way you would in Spanish. In English, if you’re not confident enough, they’ll think you’re not competent”* . Participants noted that assertive self-promotion—often required in grant writing—was not common or comfortable in their academic cultures. For example, while U.S. funders may expect strong optimism, U.K.-based funders might penalize what they interpret as exaggeration. These nuances complicate the crafting of proposals for international audiences. | Participants 1, 2, 3, 4, 5 |
|  | Perceived bias from Global North funders | Participants described skepticism towards Latin American science based on geography or institutional affiliation: *“When they see a Latin American surname and an institute from here, they already assume we don’t have the money or the resources to do what we’re proposing”* | Participant 3 |
|  | Unrealistic expectations | Participants also discussed unrealistic expectations embedded in some international calls, such as assumed access to advanced infrastructure or certain academic credentials. These requirements, while standard in some contexts, can be exclusionary when applied globally: *“In our country, few people have PhDs, but there are many with PhD-equivalent experience.”* | Participant 4 |
|  | Barriers to international networking and visibility | Another major barrier was limited access to influential networks and visibility within international academic and funding circles. The lack of funding to attend conferences was cited as a factor that reduces networking opportunities and recognition (participants 2, 4). Familiarity between reviewers and applicants—common in the Global North—was perceived to play an implicit but powerful role in evaluation outcomes (Participants 1, 2, 3, 5). | Participants 1, 2, 3, 4, 5 |
| Recommendations for More Equitable Funding Practices | Expanding Inclusion of LMICs and Mentorship Opportunities | Importance of broadening calls that explicitly include Low- and Middle-Income Countries (LMICs). They also highlighted mentorship initiatives, such as observer roles in selection panels, as effective ways to build capacity and familiarity with international standards. | Participant 5 |
|  | Valuing Comparative Advantages and Context-Specific Knowledge | Participants pointed to the Global South’s comparative advantages, including access to rich biodiversity and unique genetic resources. They also added that recognizing context-specific knowledge as a valuable contribution is crucial for fostering equitable international research collaborations. | Participants 3 and 4 |
|  | Training Tailored to the Latin American Context | Participants underscored the need for capacity-building initiatives that are practical, collaborative, and context-sensitive. Suggested areas included: (a) Project writing and narrative construction skills; (b) Interdisciplinary and collaborative project design; (c) Familiarity with administrative processes and standard requirements; (d) Real-life examples and peer feedback opportunities; (e) Hands-on exercises, including simulated panel reviews. | Participants 2, 3, 4, 5 |
