# Supplementary material for: Perceived barriers for accessing international research funding among Latin American researchers
Source: PLoS One. 2026 Apr 20;21(4):e0343593. doi: 10.1371/journal.pone.0343593 (PMC13094988; doi:10.1371/journal.pone.0343593)
Supplement: Attachment — Submitted filename: Review of manuscript PONE-D-25-50701.pdf [file pone.0343593.s002.pdf]

Manuscript: PONE-D-25-50701

Title: Barriers for accessing international research funding

Reviewer: Rafael Anta

General Comment

Congratulations to the research team. The topic is essential for Latin American science, and the paper addresses it with rigor and relevance. The mixed-methods approach is well conceived and executed, producing high-quality insights and practical recommendations. The manuscript makes a valuable contribution to understanding the barriers Latin American researchers face in accessing international funding, as ultimately, we all aspire to see a greater number of Latin American researchers securing international funding and contributing more visibly to the global scientific community.

For clarity, my observations are grouped into two areas: one concerning the contextual framing of the introduction and another addressing the use of terminology around equity and fairness.

Area 1: Contextualization of the Introduction

The paper would benefit from opening with a broader overview of how science operates in the 21st century, to situate the reader before narrowing to the Latin American perspective. Moving from a frame of "this is an inequity that must be corrected" to "this is the competitive landscape of global science, and here is how Latin American researchers are structurally disadvantaged within it" would strengthen the rationale for the study and clarify the interpretation of its results.

It could cover aspects such as the following:

- Science as a globalized enterprise that largely operates in English and functions through international knowledge networks.
- Researchers from wealthier countries benefit from greater resources, infrastructure, institutional support, and network participation, while those in Latin America face multiple structural limitations stemming from weaker and less well-funded national science systems.
- Public R&D funding is designed to serve national goals (economic competitiveness, innovation, social development, etc.), and international R&D funding, when it involves public money, is driven by strategic (not equity) objectives.
- The number of researchers competing for limited funds continues to grow faster than the volume of available resources; access to international funding will therefore become increasingly competitive.
- Grant writing is a specialized skill, one that even researchers in the Global North must deliberately cultivate, supported by professional structures developed for this purpose.

This broader framing would make the discussion of barriers and recommendations more compelling and intellectually anchored in the realities of global science.

Area 2: Use of Terminology and Framing of Equity and Fairness

The use of terms such as "financial inequity", "equitable access", and "fairness" should be reconsidered for precision and realism. International funding is not, and has never been, primarily

an instrument for global redistribution or equity. Framing it as such can weaken the argument. The concepts of asymmetry, disparity, or imbalance might better capture the dynamics at play without implying moral obligation or entitlement.

Detailed comments:

- Access to international research funding is merit-based, not equity-based; calls are not designed to ensure equal opportunity across countries, institutions, or researchers, but to support the most competitive proposals. The issue is therefore not the distribution of resources but rather how structural imbalances in national systems affect researchers' competitiveness.
- The authors could highlight that some funders are beginning to recognize their potential role in promoting a more equitable and diverse global research ecosystem (for example, Chan Zuckerberg Initiative, National Geographic Society, ...). This would allow the paper to frame "equity" as an emerging and desirable aspiration for progressive funders, rather than as a fundamental failure of the current system.
- Several challenges described in the paper, such as limited infrastructure or institutional weakness and red tape, stem from domestic science policy gaps that cannot realistically be solved through modifications in international funding design.
- The expression "*misalignment between donor priorities and local challenges*" should be revised; "differences between donor priorities and local challenges" would be more accurate, as donor priorities are defined according to their own strategic interests.
- When referring to "*recommendations for more equitable funding practices*", clarify who the intended audience is (international funders, national agencies, or research institutions?) since each operates with different mandates and responsibilities.
- If the authors wish to emphasize the importance of diversity in science (which it is), they could suggest local and international mechanisms to promote it (e.g., mentorship schemes, scholarships for underrepresented groups, etc) but I think this was not the focus of the study.
